# pH and Thrombin Concentration Are Decisive in Synthesizing Stiff, Stable, and Open‐Porous Fibrin‐Collagen Hydrogel Blends without Chemical Cross‐Linker

**DOI:** 10.1002/adhm.202203302

**Published:** 2023-01-13

**Authors:** Mattis Wachendörfer, Eva Miriam Buhl, Ghazi Ben Messaoud, Walter Richtering, Horst Fischer

**Affiliations:** ^1^ Department of Dental Materials and Biomaterials Research RWTH Aachen University Hospital Pauwelsstrasse 30 52074 Aachen Germany; ^2^ Electron Microscopy Facility Institute of Pathology RWTH Aachen University Hospital Pauwelsstrasse 30 52074 Aachen Germany; ^3^ Institute of Physical Chemistry RWTH Aachen University Landoltweg 2 52074 Aachen Germany; ^4^ Physical Chemistry DWI–Leibniz Institute for Interactive Materials Forckenbeckstr. 50 52074 Aachen Germany

**Keywords:** cross‐linking mechanisms, hydrogels, interpenetrating networks, microstructures

## Abstract

Fibrin‐collagen hydrogel blends exhibit high potential for tissue engineering applications. However, it is still unclear whether the underlying cross‐linking mechanisms are of chemical or physical nature. It is here hypothesized that chemical cross‐linkers play a negligible role and that instead pH and thrombin concentration are decisive for synthetizing blends with high stiffness and hydrolytic stability. Different fibrin‐collagen formulations (pure and with additional transglutaminase) are used and the blends’ compaction rate, hydrolytic stability, compressive strength, and hydrogel microstructure are investigated. The effect of thrombin concentration on gel compaction is examined and the importance of pH control during synthesis observed. It is revealed that transglutaminase impairs gel stability and it is deduced that fibrin‐collagen blends mainly cross‐link by mechanical interactions due to physical fibril entanglement as opposed to covalent bonds from chemical cross‐linking. High thrombin concentrations and basic pH during synthesis reduce gel compaction and enhance stiffness and long‐term stability. Scanning electron microscopy reveals a highly interpenetrating fibrous network with unique, interconnected open‐porous microstructures. Endothelial cells proliferate on the blends and form a confluent monolayer. This study reveals the underlying cross‐linking mechanisms and presents enhanced fibrin‐collagen blends with high stiffness, hydrolytic stability, and large, interconnected pores; findings that offer high potential for advanced tissue engineering applications.

## Graphical abstract

The underlying cross‐linking mechanisms of fibrin‐collagen hydrogel blends are still unclear. It is shown that thrombin concentration and pH control during synthesis are decisive for fabricating stiff, stable, and open‐porous fibrin‐collagen blends. Herein, it is revealed that chemical cross‐linkers are negligible and that the cross‐linking is based on physical fibril entanglement rather than chemical cross‐linking, creating interpenetrating, yet open‐porous fibrous networks.

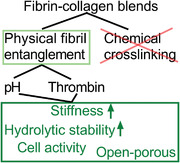

## 1 Introduction

In tissue engineering and in vitro modeling, the native extracellular matrix (ECM) is often substituted with hydrogel blends to create a biomimetic and cell‐supportive environment for cells.^[^ ^1^, ^2^ ^]^ The immediate microenvironment of cells affects their responsive cellular behavior and is a major factor for cell function regulations.^[^ ^3^, ^4^, ^5^ ^]^ Next to nutrition access and semiochemical supply, the ECM substitute's stiffness, microstructure, and stability during cultivation influence cellular responses and are essential for creating, influencing, and tailoring functional, biomimetic in vitro models. Stiffness controls cell migration, differentiation and morphology through cell–substrate interactions and anchoring.^[^ ^6^, ^7^, ^8^, ^9^ ^]^ The ECM substitute stiffness is directly associated with hydrogel microstructure, which regulates the diffusion of nutrients and removal of metabolic waste products, and also determines cell spreading and morphology.^[^ ^1^, ^10^ ^]^ For functional in vitro models, a period of cellular maturation is required after the initial biofabrication process in order to facilitate cell proliferation and differentiation, promoting cell‐to‐cell interactions, and expression of key markers.^[^ ^11^ ^]^ Hence, both the short‐ and long‐term hydrolytic stability of the hydrogels during cultivation are essential.^[^ ^12^, ^13^ ^]^ The short‐term hydrolytic stability corresponds to the degree of swelling or compaction hydrogels exert when initially infused with cell culture medium and should remain as low as possible. During long‐term cultivation, the hydrogel is degraded by encapsulated cells such as fibroblasts often found in the native extracellular matrix or by endothelial cells, which line vessel lumens and determine the vascularization of in vitro models.^[^ ^14^ ^]^ Therefore, hydrogel degradation must be controlled as cells lose their anchoring scaffold and cytotoxic byproducts can affect cell viability and functionality.^[^ ^15^, ^16^ ^]^ Live cell imaging and in situ observation of cellular processes is facilitated by hydrogels with optical transparency, however this is often described more as a helpful asset rather than an essential feature.^[^ ^12^ ^]^

A frequently used biomaterial for ECM substitutes featuring this helpful asset is fibrin, which is known for its key role in the clotting cascade. Fibrin appears either opaque or transparent depending on the fiber self‐assembly and fiber lateral size, which affect the mechanical properties and are determined by the pH during synthesis as well as the calcium and thrombin concentration.^[^ ^17^, ^18^ ^]^ Fibrin provides advantageous properties for tissue engineering applications, such as cell binding sites and facilitation of controlled release of growth factors.^[^ ^19^, ^20^, ^21^ ^]^ On the other hand, high rates of compaction (often referred to as shrinking or contraction) and fast degradation, especially in low‐concentrated fibrin hydrogels, limits use in long‐term 3D cell culture.^[^ ^22^, ^23^ ^]^

Another common biomaterial used in biomimetic ECM substitutes is collagen type I, which is the major component of natural ECM.^[^ ^24^, ^25^ ^]^ Collagen is a turbid biomaterial known for its outstanding biomimetic properties but it exhibits low stiffness and fast degradation kinetics combined with high compaction rates.^[^ ^12^, ^26^, ^27^ ^]^ As a result of its acidic origin, collagen polymerizes physically due to shifts to neutral or basic pH values and exposure to physiological temperature, both of which influence the final fiber formation and thickness.^[^ ^28^, ^29^ ^]^ Hence, for processing, collagen is usually cooled first.^[^ ^12^, ^26^ ^]^ Previous studies have used fibrin‐collagen blends in order to improve mechanical properties and reduce gel compaction.^[^ ^30^, ^31^ ^]^

As fibrin‐collagen blends have been proven to support the proliferation and migration of encapsulated cells and angiogenesis, they are frequently used in the field of tissue engineering for in vivo and in vitro models.^[^ ^32^, ^33^ ^]^ Both fibrinogen and collagen type I provide the tripeptide arginine–glycine–aspartate sequences (RGD motifs) that facilitate cell adhesion.^[^ ^34^ ^]^ Despite the great potential of fibrin‐collagen blends for tissue engineering applications, few studies focus on unraveling the underlying mechanisms of fibril formation within the co‐gels. It has not yet been clarified whether interactions between fibrin and collagen fibrils are due to chemical interactions such as the formation of new covalent bonds, or mechanical interactions, such as physically entangled fibrils and molecules.^[^ ^35^ ^]^ Early studies disagreed about the formation of chemical bonds between collagen and fibrin due to the active participation of transglutaminase factor XIII.^[^ ^36^, ^37^, ^38^ ^]^ Transglutaminase, best known for its stabilization function for fibrin during blood clotting, catalyzes the formation of covalent bonds between free *ε*‐amino groups of lysine from a protein or peptide bond and *γ*‐carbonyl groups of deamidated glutamine proteins or peptide‐bound y‐carboxamide groups.^[^ ^39^, ^40^ ^]^ As opposed to its mammalian counterpart, the activity of microbial transglutaminase is independent of calcium and stable for a broader range of temperatures and pH.^[^ ^41^ ^]^ Recent studies focusing on or using fibrin‐collagen blends utilized synthesis protocols with transglutaminase^[^ ^32^ ^]^ or without transglutaminase.^[^ ^33^, ^42^ ^]^ A broad range of studies has neglected the control of the pH of fibrinogen solution during hydrogel blend synthesis^[^ ^35^, ^43^, ^44^ ^]^ even though fibrin formation is highly susceptible to pH shifts.^[^ ^45^, ^46^, ^47^ ^]^ However, knowledge about the effect of reaction conditions (pH, temperature) during hydrogel synthesis, the active participation of each ingredient, and the underlying fibril formation mechanisms will be essential for designing ECM substitutes with high biofunctionality and biomimicry, as well as for understanding and predicting multifactorial material behavior in terms of mechanical properties, hydrolytic stability, and cellular responses.

In this study, we hypothesized that the cross‐linking of fibrin‐collagen blends is predominantly based on physical interactions rather than chemical cross‐linking and the formation of new covalent bonds. We further hypothesized that thrombin concentration and pH control during hydrogel synthesis are powerful tools to the fabrication of advanced fibrin‐collagen hydrogel blends.

Hence, we assessed the role of exogenous transglutaminase in fibrin‐collagen blends and the effects of thrombin concentration on the final blends’ mechanical properties, hydrolytic stability, and hydrogel microstructure. We further highlight the importance of pH control during hydrogel synthesis and combine the results to create advanced fibrin‐collagen blends exhibiting great compressive strength, reduced compaction, and long‐term stability during incubation, and an open‐porous microstructure, while supporting proliferation of endothelial cells.

## 2 Results

In the current study, we selected the fibrin and collagen concentration based on previous studies focusing on fibrin‐collagen blends with identical raw products to ensure a relevant comparison.^[^ ^32^ ^]^ The transglutaminase concentration was adjusted to correspond to commonly used concentrations, for example, 0.2–0.4 mg per mg fibrinogen used for enzymatic cross‐linking.^[^ ^48^, ^49^ ^]^ Tranexamic acid concentration was similar to previous studies,^[^ ^49^ ^]^ however we would like to emphasize that blends synthetized without tranexamic acid showed similar degradation kinetics in preliminary studies, as also elucidated further below. Thrombin concentrations of 0.06–0.185 U per mg fibrinogen were chosen due to practical reasons, as much lower or much higher concentrations would result in hydrogels without sufficient stability for reliable handling and cross‐linking times insufficient for reliable processing, respectively. The concentrations are within ranges of regularly used mixtures, which can range from 0.001 to 20 U thrombin per mg fibrinogen, but are usually around 0.1 to 0.2 U thrombin per mg fibrinogen.^[^ ^31^, ^32^, ^48^, ^49^, ^50^ ^]^ Concentration of calcium chloride were chosen based on preliminary studies, whereas a much lower concentration resulted in fibrin‐collagen blends with reduced stability while increased concentrations would result in inhomogeneous fibrin clotting and reduced cellular adhesion, respectively. Calcium chloride concentration for fibrin gels was adjusted to ensure optical clarity. Calcium chloride concentrations in previous studies can range from 0.006 to 1.1 mg per mg fibrinogen,^[^ ^45^ ^]^ whereas more commonly used concentrations range from 0.02 to 0.08 mg mL^−1^ fibrinogen,^[^ ^32^, ^48^, ^49^ ^]^ which corresponds to our applied concentrations as well.

### 2.1 In Vitro Swelling and Degradation of Non‐Cell‐Laden and Cell‐Laden Hydrogels

**Figure 1:**
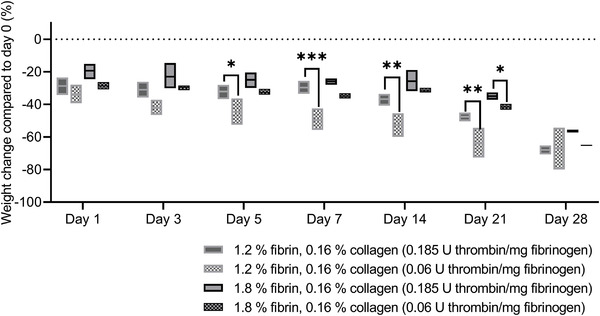
In vitro hydrolytic degradation of 1.2% fibrin with 0.16% collagen and 1.8% fibrin with 0.16% collagen blends. Fibrin‐collagen blends and fibrin were synthetized at pH 8 and pH 7.4, respectively, without additional transglutaminase. Initial shrinkage and degradation were reduced with higher thrombin concentration.

Fibrin‐collagen blends composed of 1.2% (12 mg mL^−1^) and 1.8% (18 mg mL^−1^) fibrin with 0.16% (1.6 mg mL^−1^) collagen initially shrank to a lesser degree and provided lower weight loss during hydrolytic degradation in PBS when cross‐linked with 0.185 U thrombin compared to 0.06 U thrombin per mg fibrinogen (**Figure** **1**). Significant differences were found on days 5, 7, 14, and 21 for 1.2% fibrin with 0.16% collagen and on day 21 for 1.8% fibrin with 0.16% collagen. The blends with higher fibrin concentration provided both a lower initial shrinking rate and weight loss during the first 21 days of hydrolytic degradation.

**Figure 2:**
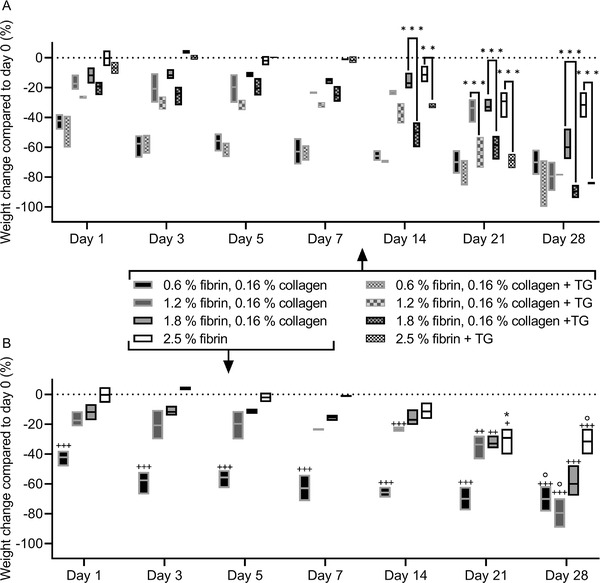
In vitro hydrolytic degradation of fibrin‐collagen blends and fibrin hydrogel. All fibrin‐collagen blends and fibrin were synthetized at pH 8 and pH 7.4, respectively, and with a thrombin concentration of 0.185 U thrombin per mg fibrinogen. A) Initial shrinkage and degradation increase with the addition of transglutaminase (TG). B) Initial shrinkage decreases with fibrin concentration. All blends were stable for at least 21 days. Significant weight changes compared to day 0 are marked with ++/+++, while significant weight changes to the previous time point are marked with *. Dissolved samples are marked with.

For 0.6% (6 mg mL^−1^) fibrin with 0.16% collagen blends synthetized without or with exogenous transglutaminase, no statistical differences were found between weight losses during hydrolytic degradation (**Figure** **2**A). Blends composed of 1.2% fibrin with 0.16% collagen without transglutaminase showed decreased initial shrinking and lower weight losses during incubation compared to identical blends synthetized with transglutaminase. Significant differences in weight loss were found on day 21. A similar pattern was recorded for 1.8% fibrin with 0.16% collagen blends, as initial shrinking and weight loss increased due to the addition of transglutaminase. The weight loss of the blend with transglutaminase was significantly higher to its counterpart without transglutaminase from day 14 onwards. As opposed to fibrin‐collagen blends, the 2.5% (25 mg mL^−1^) fibrin hydrogel showed no initial weight loss and retained its original weight until day 14. From day 14 to day 28, the 2.5% fibrin with additional transglutaminase showed a significantly higher weight loss than fibrin gels with transglutaminase.

The following results are focused on the fibrin‐collagen blends and the pure fibrin hydrogel synthetized without transglutaminase, as they were used for both the compression test and the human umbilical vein endothelial cells (HUVECs) proliferation study (Figure 2B). The 0.6% fibrin with 0.16% collagen blend showed a significant weight loss of 42.12 ± 5.41% on day 1 compared to the day of synthesis. The weight of the blend progressively decreased throughout the experiment without significant changes, while one sample had dissolved on day 28, resulting in a final weight loss of 70.02 ± 11.96%. The 1.2% fibrin with 0.16% collagen blend initially lost 17.33 ± 5.36% of weight up to day 1, however, no significant initial weight loss was recorded. The weight loss remained constant but progressed from day 14 onwards, as the blend provided a significantly lower weight on day 14 (−22.91 ± 1.84%) and day 21 (−33.66 ± 8.53%) compared to the day of synthesis. On day 28, the blend had lost a significant 79.43 ± 13.85% of the initial weight with one dissolved sample. The 1.8% fibrin with 0.16% collagen blend provided a constant weight loss between 11.73 ± 5.41% on day 1 and 15.23 ± 2.18% on day 14. The weight losses on days 21 (32.93 ± 4.69%) and 28 (59.96 ± 10.97%) represented a significant change compared to the initial weight and no sample dissolved. Similarly, 2.5% fibrin hydrogels provided no significant weight loss compared to the initial weight from day 1 (0.27 ± 5.25%) to day 14 (11.35 ± 5.57%). However, the blend lost significant weight on day 21 (29.13 ± 9.65%) and leveled off at a weight loss of 31.59 ± 12.06% with one dissolved sample on day 28. The pH of the PBS used for hydrolytic degradation leveled off between 6.75 and 7 for all samples within the first 21 days and then rose with progressing degradation to 7.05–7.35 on day 28 (Figure S3, Supporting Information).

### 2.2 Gel Opacity and Compressive Strength

**Figure 3:**
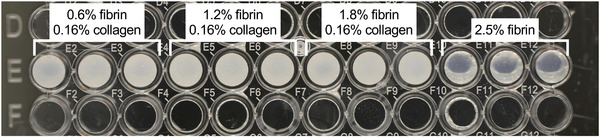
Opacity and transparency of fibrin‐collagen blends and fibrin. All fibrin‐collagen blends and fibrin were synthetized at pH 8 and pH 7.4, respectively, without additional transglutaminase and with a thrombin concentration of 0.185 U thrombin per mg fibrinogen. Fibrin‐collagen blends appear turbid, while pure fibrin appears transparent. Quantification of turbidity can be found in Figure S1, Supporting Information.

All fibrin‐collagen blends appeared turbid, while pure fibrin gels were optically transparent (**Figure** **3**). Quantification of optical appearance can be found in Figure S1, Supporting Information.

**Figure 4:**
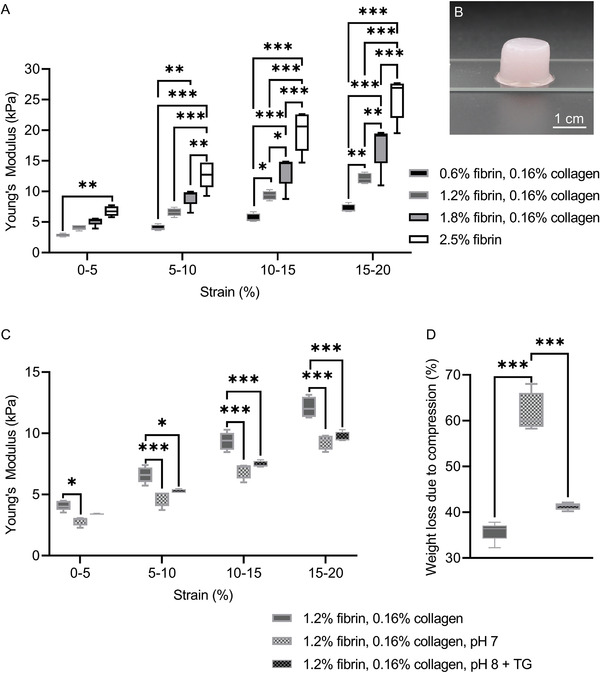
A) Compressive strength of fibrin‐collagen blends and fibrin synthetized without transglutaminase and at pH 8 and pH 7.4, respectively. B) Exemplary sample of 1.2% fibrin with 0.16% collagen blend used for compression tests, ≈14 mm in diameter and 12 mm in height. C) Compressive strength of 1.2% with 0.16% collagen blends decreased when synthetized with fibrinogen solution at pH 7 instead of pH 8 and with the addition of transglutaminase (TG) (application time 6 h at 4 °C and pH 8 prior to thrombin cross‐linking). D) In contrast to (C), the weight loss due to compression increased. For all blends, a concentration of 0.185 U thrombin per mg fibrinogen was used.

The fibrin‐collagen blends provided Young's moduli at 0–5% strain of 2.82 ± 0.25, 4.09 ± 0.4, and 5.11 ± 0.67 kPa, with increasing fibrin mass fraction and 6.78 ± 0.8 kPa for pure fibrin gels (**Figure** **4**A). Significant differences between the gel groups were found at each strain region and increased with higher strain rates. The compressive strength of the blends increased to a greater extent at higher strain regions, as the 1.8% fibrin‐collagen and the pure fibrin gel provided compressive strengths of 17.45 ± 3.65 and 25.23 ± 3.44 kPa, respectively, at 15–20% strain. Correspondingly, the two lowest concentrated fibrin‐collagen blends provided a compressive strength of 7.18 ± 0.68 and 12.13 ± 0.83 kPa. In general, the weight loss due to compression decreased significantly with increasing fibrin concentration and was highest at 0.6% fibrin with 0.16% collagen blends at 74.65 ± 2.82% and lowest at 2.5% fibrin hydrogel at 10.41 ± 2.76% (Figure S2, Supporting Information).

Fibrin‐collagen blends synthetized with fibrinogen at pH 7 or with additional transglutaminase, cooled to 4 °C at pH 8 for 6 h prior to collagen addition, provided significantly lower compressive strength compared to blends of identical fibrin‐collagen fraction synthetized at pH 8 without transglutaminase (Figure 4C). The differences increased with increasing strain. The weight loss due to compression of the blend synthetized at pH 7 was significantly higher at 61.60 ± 4.37% compared to the weight loss of the other two blends at 41.34 ± 0.79% and 35.82% ± 2.09% (Figure 4D). Over the course of 7 days of hydrolytic degradation in PBS, with ≈60% of its initial Young's modulus, the 1.2% fibrin with 0.16% collagen blend retained significantly more stiffness compared to the identical blend with additional transglutaminase (Figure S4, Supporting Information) at 50%. The 1.2% fibrin with 0.16% collagen blend synthetized with fibrinogen solution at pH 7 was too soft to be reliably measured after 7 days of hydrolytic degradation.

### 2.3 Hydrogel Microstructure

**Figure 5:**
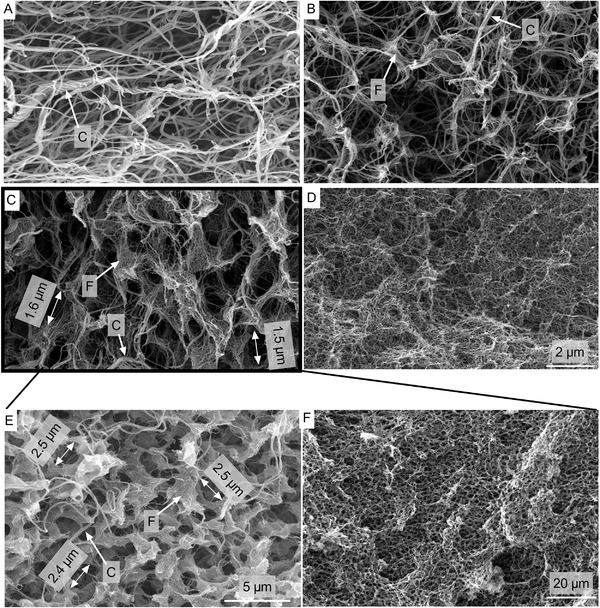
SEM pictures of fibrin‐collagen blends and fibrin. All fibrin‐collagen blends and fibrin were synthetized at pH 8 and pH 7.4, respectively, without additional transglutaminase and with a thrombin concentration of 0.185 U thrombin per mg fibrinogen. A) 0.6% fibrin with 0.16% collagen. B) 1.2% fibrin with 0.16% collagen. C) With increasing fibrin fraction, a unique microstructure of fibrin‐collagen blends with honeycomb‐like features is formed, best observed in 1.8% fibrin with 0.16% collagen blends (C) at lower magnifications (micrograph [E] and [F]). Magnification of (A)–(C) identical with (D). Collagen (C) and fibrin (F) fibers seem to intertwine while coexisting individually, forming an interpenetrating network. D) Pure 2.5% fibrin gels show a typically dense microstructure.

Pure collagen gel showed an arbitrary microstructure composed of thick fibers, intertwined to coiled fiber bundles (Figure S11, Supporting Information). Fibrin‐collagen blends composed of 0.6% fibrin with 0.16% collagen showed no distinct microstructure during scanning electron microscopy (SEM) microscopy (**Figure** **5**A). Triple‐coiled collagen fibers were highly present and intertwining, forming a rather arbitrary fibrous microstructure. In 1.2% fibrin with 0.16% collagen blends, both fibrin and collagen fibers coexisted and interacted with each other and fibrous nodal points became more prominent (Figure 5B). An increasing number of distinct oval pores with sizes of ≈1–2 µm were found in 1.8% fibrin with 0.16% collagen blends (Figure 5C). Fibrin showed a more fanned out structure, while collagen fibers intertwined through the porous but interconnected microstructure. On SEM pictures with lower magnification, a honeycomb‐like microstructure was found with defined and homogenous pores (Figure 5E,F). In 2.5% fibrin hydrogels, a dense and homogenous microstructure was found with pores at the nanometer scale together with short and fine fibers (Figure 5D).

SEM images with lower magnification of all hydrogels can be found in Figure S9, Supporting Information, where the described microstructural differences are distinctly visible as well. Similar microstructures and pore morphologies were found when fibrin‐collagen blends and fibrin hydrogel were synthetized with additional transglutaminase (Figures S6 and S10, Supporting Information), indicating no active role for transglutaminase in the intertwining of collagen and fibrin fibrils.

### 2.4 Endothelial Cell Proliferation on Hydrogel Blends

**Figure 6:**
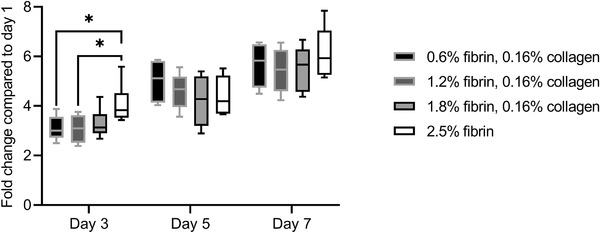
CCK‐8 assay of HUVECs seeded on fibrin‐collagen blends and fibrin hydrogel. All fibrin‐collagen blends and fibrin were synthetized at pH 8 and pH 7.4, respectively, without additional transglutaminase and with a thrombin concentration of 0.185 U thrombin per mg fibrinogen. HUVECs proliferated in equal measure on the hydrogels. Qualitative microscopy pictures can be found in Figure S7, Supporting Information.

No significant differences between cell concentrations on the different hydrogels were found on day 1 after seeding (Figure S8, Supporting Information). Compared to the two lowest concentrated fibrin‐collagen gels, HUVECs on the pure fibrin gel provided significantly higher metabolic activity on day 3, with a fold change compared to day 1 of 4.11 ± 0.79 (**Figure** **6**C). However, on days 5 and 7 no significant differences were found and the fold changes amounted to 5.66 ± 0.80 (0.6% fibrin, 0.16% collagen), 5.42 ± 0.85 (1.2% fibrin, 0.16% collagen), 5.53 ± 0.86 (1.8% fibrin, 0.16% collagen), and 6.14 ± 0.99 (2.5% fibrin). Ultimately, a dense HUVEC monolayer was formed on all ECM substitutes. Fluorescence images of HUVECs stained with calcein‐AM on the hydrogel blends and on specific days can be found in Figure S7, Supporting Information. All cell‐laden hydrogels were stable during HUVEC proliferation. The lyophilized weight of the blends did not decrease significantly over the term of 7 days (Figure S5, Supporting Information). Hence, the hydrogels were not degraded by the cells or other medium‐activated mechanisms.

## 3 Discussion

Fibrin‐collagen hydrogel blends exhibit a great potential to substitute native ECM in in vitro models with high biomimicry due to excellent cell‐supporting motifs and inherent mimetic resemblance to native tissue. However, high compaction and mediocre long‐term stability reduce the scope of potential tissue engineering applications. Moreover, the underlying cross‐linking mechanisms of fibrin‐collagen blends are still not fully elucidated as both synthesis protocols with and without chemical cross‐linkers in the form of microbial transglutaminase are used as described above. Moreover, protocols often lack pH control of individual hydrogel solutions, while fibrin‐based hydrogels are particularly sensitive to pH shifts and ionic strength gradients.^[^ ^45^, ^51^ ^]^ In this study, we investigated the effects of additional transglutaminase and thrombin concentration on the hydrolytic stability and mechanical properties of fibrin‐collagen blends. We demonstrated the importance of pH control during hydrogel synthesis and succeeded in fabricating advanced fibrin‐collagen blends compared to previous studies based on our findings.

Previous studies have shown that increasing thrombin concentration and higher final thrombin‐to‐fibrinogen ratios decrease gel compaction and enhance the mechanical properties of fibrin hydrogels, mainly due to decreasing fibrin fiber diameters at higher thrombin concentrations.^[^ ^31^, ^42^, ^52^, ^53^ ^]^ In our study, we confirmed this relation for the first time in fibrin‐collagen hydrogel blends as the compaction rate of the blends decreased at higher thrombin‐to‐fibrinogen ratios (Figure 1). This is mainly due to the previously reported mechanisms of fibrin fiber thickness alterations, which result in variations of single fiber binding rigidity, as opposed to changes in collagen I structure, which is reportedly unaffected by thrombin concentration.^[^ ^31^ ^]^ Our analysis of compaction and degradation rates differed from methods using optical measurements, methods which are inherently flawed since gel sizes are often measured only 2D.^[^ ^26^, ^42^ ^]^ In our study, we weighed the hydrogel blends and calculated the weight change on the respective days of incubation accordingly, corresponding to the degree of compaction and degradation rate, as reported previously.^[^ ^49^ ^]^ The wet weight of the hydrogels is dominated by the weight of the retained fluid and not the weight of the fibrous network, hence the weight directly corresponds to gel volume, which could be calculated using the fluid density. The shrinkage or swelling of hydrogels usually occurs within a day of immersion in fluid, also often referred to as “equilibrium swelling.” Afterward, the weight and volume of the hydrogels remains constant until polymer degradation commences, which is accompanied by weight changes and an increase of pH as shown in our previous study^[^ ^49^ ^]^ and shown in Figure S3, Supporting Information, where a significant increase in pH was measured from day 21 to day 28 due to the advancing degradation. This is also further elucidated at a later point within the discussion. Our aim was to fabricate fibrin‐collagen blends with low degrees of compaction, and all other experiments were performed with the high thrombin concentration. High thrombin concentrations have been found to enhance the proliferation of mesenchymal stem cells and dermal fibroblasts, although high fibrin concentrations have been attributed as a more decisive factor.^[^ ^54^, ^55^ ^]^ We found that the final concentration of 0.185 U thrombin per mg fibrinogen was still easy to handle, for example, for filling molds, as cross‐linking occurred within 1–2 min, while a further distinct increase of thrombin concentration led to pipette clogging and inhomogeneous hydrogel cross‐linking.

In addition to low thrombin concentrations, we also found that additional transglutaminase had adverse effects on the compaction ratio and long‐term stability of fibrin‐collagen blends and fibrin hydrogel (Figure 2). Hydrogel blends synthetized with additional transglutaminase compacted to a higher degree and subsequently provided more significant weight losses throughout incubation time. Although these results cannot categorically preclude transglutaminase‐mediated chemical cross‐linking between collagen and fibrin, these data suggest that transglutaminase did not react with the components and was therefore flushed out during the first period of incubation, resulting in a higher weight loss ratio of fibrin‐based hydrogel blends with additional transglutaminase. It is possible that the interaction with transglutaminase and CaCl_2_ led to further ion–ion interactions that increased the compaction of the hydrogel blends gels. We should mention that the used fibrinogen source has a moderate degree of purity with at least 75% of clottable protein. Further studies are necessary to investigate whether the small amounts of byproducts influenced the observed cross‐linking mechanisms. However, we would like to emphasize that the used fibrinogen is one of the most commonly used sources in the field of tissue engineering and the presented protocols ensure repeatability with this product. It must be noted that definitely a minima of transglutaminase/factor XIII is already present in the raw fibrinogen product, as fibrin formation also proceeds without exogenous transglutaminase, as shown in our pure fibrin gels and elsewhere.^[^ ^17^ ^]^ When microbial transglutaminase is used for the enzymatic cross‐linking of fibrinogen to gelatin, a pre‐incubation time of 1 h at 37 °C at physiological pH is usually added prior to thrombin cross‐linking.^[^ ^48^, ^49^ ^]^ This is impossible to incorporate during the synthesis of fibrin‐collagen blends, as collagen naturally gels already at room temperature and, due to pH shifts from acidic to basic, would already have formed a gel during pre‐incubation, preventing subsequent thrombin cross‐linking. To maintain the collagen at a liquid state, constant cooling is required, prolonging the time needed for sufficient enzymatic activity, although microbial transglutaminase is known to have high enzymatic activity within a broad temperature and pH range.^[^ ^39^, ^41^ ^]^ This would be impractical for at least cell encapsulation studies and biofabrication processes, but could be more feasible with self‐prepared instead of commercial collagen, which was shown to self‐catalyze cross‐linking due to transglutaminase within 6 h at room temperature.^[^ ^56^ ^]^ However, when we synthetized fibrin‐collagen blends with additional transglutaminase and precooled the mixture for 6 h at 4 °C to allow more time for enzymatic cross‐linking and simultaneously maintaining the mix in a liquid state, the blends provided significantly lower stiffness compared to blends synthetized without transglutaminase. This is contradictory to the assumption of an additionally chemically, and as a consequence of this denser cross‐linked hydrogel blend. Likewise, the blends with added transglutaminase retained a significantly lower degree of their initial compressive strength after 7 days of hydrolytic degradation (Figure S4, Supporting Information). Moreover, SEM images revealed an identical microstructure of fibrin‐collagen blends and fibrin both with and without exogenous transglutaminase, supporting our initial hypothesis that exogenous transglutaminase did not participate to a great extent in the fibril formation (Figure 5 and Figure S6, Supporting Information). Unreacted, hence excess, transglutaminase is likely to diffuse out from the gels within the first days, as mentioned above. It is important to mention that fibrin‐collagen blends with high cytocompatibility and cell‐supportive properties have been synthetized with additional microbial transglutaminase before.^[^ ^32^ ^]^ It is also known that transglutaminase does not affect the triple helical structure of collagen I.^[^ ^56^ ^]^ However, we would like to emphasize that microbial transglutaminase is expected to act as a weak cell adhesion molecule, as opposed to its native counterpart, due to great biochemical and physical differences, and also that it is known for its immunogenic and potentially pathogenic properties, suppressing intestinal protective barriers and being involved in celiac disease initiation.^[^ ^57^, ^58^ ^]^ Based on our observations, we concluded that additional transglutaminase is superfluous for the synthesis of fibrin‐collagen blends and fibrin hydrogels, providing a low compaction rate and high stability during incubation.

The optimized hydrogel blends without transglutaminase and with high thrombin concentration were stable for at least 21 days of incubation (Figure 2B). It must be noted that compaction and long‐term stability are also dependent on the immersion medium (e.g., type and valency of ions, ionic strength and resulting osmotic pressure).^[^ ^1^, ^59^, ^60^ ^]^ The degradation experiments were performed with non‐cell‐laden gels, hence the degradation mechanism is likely to be hydrolysis as hydrolytically unstable bonds are dissolved over time. The pH of the PBS used for immersion was constant in accordance with gel stability, indicating a stable degree of ionization during the first 21 days. As degradation progressed, the pH of the PBS changed and increased significantly (Figure S3, Supporting Information), as also reported elsewhere.^[^ ^12^, ^61^ ^]^ Fibrin is prone to proteolytic degradation by plasmin or metalloproteinases, and the degradation of the gels was possibly accelerated by plasminogen, which is present in most fibrin raw product preparations.^[^ ^62^ ^]^ As a fibrinolysis inhibitor, we added tranexamic acid during gel synthesis, but we suspect that it was flushed out during the first days, as preliminary experiments have shown no advantage of tranexamic acid on the degradation rate of the gels. For long‐term in vitro studies with cell‐laden gels, fibrinolysis inhibitors should be added directly into the immersion cell culture medium. After all, the compaction of collagen and fibrin is predominantly attributed to stresses built up after gelation and the final fibril formation. In contrast, pure collagen gels compact up to 40–80% within 1 day and fibrin gels have even compacted up to 90% of the initial volume.^[^ ^26^, ^31^, ^63^ ^]^ The compaction of fibrin‐collagen blends is mainly augmented by the presence of fibrin, which is in agreement with our studies, as compaction decreases with increasing fibrin concentration (Figure 2B).^[^ ^44^ ^]^ Compared to previous studies, our blends provided lower compaction rates and higher long‐term stability for 21 days, which is sufficient for a broad range of in vitro applications.^[^ ^11^, ^64^ ^]^

Next to the adverse effects of additional transglutaminase and low thrombin concentration, we also found that pH control or shifts, by only one unit, during hydrogel synthesis can dramatically affect the mechanical properties and degradation rate of fibrin‐collagen blends. When we adjusted the pH of the fibrinogen solution to 7 instead of 8, 1.2% fibrin with 0.16% collagen blends provided significantly lower compressive strength and significantly higher weight loss due to compression (Figure 4C,D). Moreover, as opposed to blends synthetized at pH 8, which retained most of their initial stiffness, blends synthetized at pH 7 were too soft to be measured using unconfined compression tests after 7 days of hydrolytic degradation (Figure S4, Supporting Information). Fibrin forms a firm gel at various pH ranges between 6.5 and 8.5 or 9 as reported previously, although it is more prone to shift to more acidic than basic pH ranges.^[^ ^45^, ^65^, ^66^ ^]^ Next to pH, ionic strength is also crucial for the formation of stable fibrin hydrogels, and we used a final CaCl_2_ concentration of 20 mm, which has been used to fabricate stable and transparent pure fibrin gels before.^[^ ^45^, ^66^ ^]^ Collagen, on the other hand, displays higher pH dependencies and is known to form more rigid and thicker fibers with stronger inter‐fiber attractive forces (larger net‐electrostatic forces due to changes in the ionization of amino acid side chains), resulting in higher compression modulus and overall stability when prepared at basic pH in contrast to slightly acidic or neutral pH.^[^ ^67^, ^68^, ^69^, ^70^ ^]^ Hence, the drop of compressive strength and hydrolytic stability of fibrin‐collagen gels prepared with fibrinogen solution with pH 7 instead of pH 8 is attributed to the attenuation of collagen and not fibrin. It is possible that as a result to the intertwining of collagen and fibrin, fibrin fibrils were as well significantly weakened. Although the mechanical properties of collagen‐fibrin blends are mainly dominated by fibrin content, as shown by us (Figure 4A) and others,^[^ ^42^, ^71^ ^]^ based on our study we would like to emphasize that the control and regulation to basic pH are important for the fabrication of fibrin‐collagen blends with enhanced mechanical properties and high stability.

The optimized fibrin‐collagen blends and fibrin presented in this study provide distinctly enhanced stiffness (Figure 4A), sometimes tripling or even quadrupling the Young's moduli compared to studies working with similar hydrogel compositions or studies with higher protein content.^[^ ^32^, ^42^ ^]^ Likewise, the weight loss due to compression was reduced (Figure S2, Supporting Information). Fibrils rotate in the direction of stretch, and subsequently, these network stresses are transduced into fluid pressure, prompting fluid flow through the poroelastic material and resulting in liquid loss during compression.^[^ ^72^, ^73^ ^]^ The mechanical properties of fibrillar‐based hydrogels are mainly dependent on the single fiber intrinsic properties (binding rigidity, flexibility, and length) and their organization in the resulting network, namely, entanglement length (distance between entanglement points) or cross‐linking points, and mesh size (the average spacing between fibers).^[^ ^17^, ^47^, ^59^, ^74^, ^75^, ^76^ ^]^ The SEM pictures of the fibrin‐collagen blends revealed a unique, honeycomb‐like microstructure with large (up to 2 µm), interconnected pores at higher fibrin concentrations (Figure 5C,E,F). Previously, pores in fibrin gels were considered large with a pore size of 1 µm in SEM pictures,^[^ ^73^ ^]^ smaller compared to the pores shown in the mentioned pictures. Collagen and fibrin fibers were distinguished by SEM pictures of pure collagen and fibrin gel, respectively, where collagen fibers are present as thick fibers which are coiled to characteristic fiber bundles of 0.15–0.2 µm, while fibrin fibers are much smaller, thinner, and wispier (Figure 5 and Figure S11, Supporting Information). At low fibrin concentrations, collagen fibrils were dominant, and with increasing fibrin concentration fibrin‐typical, wispy regions formed at specific domains leaving round and oval pores, indicating an open‐porous but interconnected network. To our knowledge, this is the first time that such a microstructure has been observed in fibrin‐collagen blends. Pure fibrin gels provided a dense microstructure typical for fibrin, similar to previous studies.^[^ ^31^, ^45^, ^47^ ^]^ In our fibrin‐collagen blends, collagen and fibrin formed independent networks with individual fibrils that are highly entangled and intertwined in a serial‐like manner, consistent with previous studies focusing on fibrin‐collagen blends.^[^ ^35^ ^]^ Hence, they form an interpenetrating polymer network, which is defined as an entangled network form of polymers without chemical bonds, whereas at least one polymer is synthetized and/or cross‐linked in the presence of the other.^[^ ^77^ ^]^

Although from SEM analysis it cannot be directly concluded that interactions between fibrin and collagen are of a physical rather than chemical nature, the data suggest that predominantly physical interactions in the form of fibril entanglements are present, as the addition of chemical cross‐linker transglutaminase did not change the hydrogel microstructure, as mentioned above (Figure 5 and Figure S6, Supporting Information). This supports our initial hypothesis. Our fibrin‐collagen blends provide a more open‐porous microstructure compared to other studies, which report pore sizes much smaller than 1 µm,^[^ ^31^, ^44^, ^64^ ^]^ which is generally favorable for cell encapsulation applications but adverse for mechanical strength. Although the distinct pores forming at higher fibrin concentrations decrease mechanical strength, simultaneously the crucial density of branching points is increased as distinct polymer nodes formed. Consequently, the overall mechanical strength of the blend is increased and fluid loss due to compression decreases. As mammalian transglutaminase/factor XIII is present in the raw fibrinogen product as mentioned above, a minor contribution of exogenous transglutaminase cross‐linking in the resulting hydrogel properties should not be excluded. However, the mechanical properties of the formed blends are mainly driven by collagen and fibrin fibers co‐entanglements, resulting in a strong interpenetrating polymer network. We would also like to emphasize that in contrast to previous studies that have reported a more dense microstructure with thinner fibers and smaller pores at higher thrombin concentrations,^[^ ^31^, ^44^ ^]^ we observed an open‐porous microstructure with large pores in our final blends (Figure 5C,E,F), indicating that high thrombin concentrations and an open‐porous microstructure are indeed compatible with each other.

HUVECs proliferated on all fibrin‐based gels and formed monolayers within 7 days (Figure S7, Supporting Information), showing their expected and well‐known cell‐supporting properties. We chose HUVECs as an exemplary but highly relevant cell type in the field of tissue engineering and in vitro models to confirm that the high cell compatibility of fibrin‐collagen blends is also applicable for our synthesis protocols. The blends were stable during cell proliferation (Figure S5, Supporting Information) in contrast to other studies reporting rapid HUVEC‐mediated hydrogel degradation.^[^ ^11^, ^78^ ^]^ This confirms the suitability of our synthesis approach for long‐term, cell‐laden applications. Our method does not distinguish between degraded ECM and produced ECM by the endothelial cells. However, if bulk degradation of the gels occurred, it is very unlikely that this was compensated by ECM remodeling of HUVECs at the same rate. Previous studies have reported a 60% weight loss due to endothelial activity within 7 days,^[^ ^11^ ^]^ and as the presented data shows constant dry weight, we conclude that no bulk degradation of the hydrogels occurred. Encapsulation of cells like smooth muscle cells, fibroblasts, or mesenchymal stem cells should be highly compatible with our fibrin‐collagen blends, as these cells have been encapsulated in similar blends before and show high viability and characteristic stretching within more dense hydrogel networks such as collagen‐agarose, fibrin, or pure collagen.^[^ ^32^, ^71^, ^79^ ^]^ Cell‐mediated bulk degradation of fibrin can be reduced by adding protease inhibitors such as aprotinin, aminocaproic acid, and tranexamic acid, as mentioned above and elsewhere.^[^ ^62^, ^66^, ^80^, ^81^ ^]^ However, local and partial fibrin degradation by metalloproteinases favors the development of extracellular and pericellular matrices, cell penetration and anchoring, and facilitates smooth muscle cell proliferation, invasion, tubulogenesis, and vascularization in general.^[^ ^43^, ^52^, ^82^, ^83^, ^84^, ^85^ ^]^ For cell migration, spaces between fibrils and pores must be created by the degradation of ECM or must be large enough at the outset.^[^ ^86^, ^87^ ^]^ Thus, our fibrin‐collagen blends with distinct pores could be especially promising for further cell invasion, migration, chemotaxis, angiogenesis, or motility studies: In SEM pictures they provide pores of ≈2 µm (Figure 5C). The preparation of hydrogel samples for SEM is expected to result in substantial shrinkage, resulting in smaller fibril diameters and void spaces due to the fixation in glutaraldehyde followed by dehydration and high vacuum during SEM.^[^ ^88^, ^89^ ^]^ Thus, the actual pore sizes of the gels are expected to be even larger than depicted in SEM images. Pore sizes of commercial transwell inserts used for the proposed studies focusing on small cells are 3 or 8 µm (e.g., for endothelial cells, neuronal cells, leukocytes), underlining the potential of the fibrin‐collagen gels.

## 4 Conclusion

In our study, we investigated the effect of thrombin concentration and additional exogenous transglutaminase on the mechanical properties and hydrolytic stability of fibrin‐collagen blends and fibrin hydrogels. Low thrombin concentrations and the addition of transglutaminase had adverse effects on gel compaction and stiffness. Together with SEM analysis, we revealed that mechanical interactions in the form of physical fibril entanglement, and not enzymatically activated chemical interactions between collagen and fibrin, dominate. Moreover, we showed that pH control and regulation to basic regimes during hydrogel synthesis is key for the fabrication of stable hydrogels with excellent mechanical properties. Our final protocols resulted in fibrin‐collagen blends with increased stiffness, reduced compaction, and improved in vitro hydrolytic stability. In addition, we introduced a blend that exhibits a unique microstructure with distinct, large pores and which is therefore promising for improved cell support in tissue engineering applications.

## 5 Experimental Section

### Hydrogel Stock Solution Preparation

Collagen from calf skin (Collagen G, L7213, Sigma‐Aldrich, Darmstadt, Germany) was synthetized according to the manufacturer's protocol. Briefly, 0.7 m NaOH and 1 m HEPES buffer (P05‐01100, PAN‐Biotech GmbH, Aidenbach, Germany) were mixed equally (solution A). This mixture was then mixed equally with 10× DMEM (F0455, Biochrom, Berlin, Germany) and the pH of the solution was adjusted between 7.9 and 8.05 (solution B) using 0.7 m NaOH. Both the Collagen G and the solution B were kept cool at 4 °C and mixed at a ratio of 8:2 to produce the ready‐to‐use solution for gelling, resulting in a final collagen concentration of 3.2 mg mL^−1^. Prior to use, this solution was kept cool and used within 30 min after synthesis. Fibrinogen from bovine plasma (F8630, Sigma‐Aldrich, Darmstadt, Germany) was dissolved with 50 mg mL^−1^ in PBS by stirring at 37 °C for 24 h. The fibrinogen solution was then sterile filtered (431218, Corning, Arizona, USA), aliquoted, and frozen at −20 °C. Thrombin from bovine plasma (SRP6556, Sigma‐Aldrich, Darmstadt, Germany) was diluted in PBS to 100 U mL^−1^, aliquoted, and frozen at −20 °C. Microbial transglutaminase (SKU: 5060341114533, Special Ingredients, Chesterfield, UK) was dissolved in PBS with 60 mg mL^−1^ for 6 h at 37 °C and subsequently sterile filtered (0.2 µm sterile Syringe Filter, Filtropur S, Sarstedt, Nürnbrecht, Germany), aliquoted, and frozen at −20 °C. Calcium chloride (CaCl_2_, CN93.2, Carl Roth, Karlsruhe, Germany) was dissolved in Millipore water with 50 mg mL^−1^ (450 mm), sterile filtered in the same manner, aliquoted, and frozen at −20 °C.

### Preparation of Fibrin‐Collagen Hydrogel Blends and Fibrin Hydrogel

Fibrin‐collagen blends were prepared with final fibrinogen concentrations of 0.6%, 1.2%, and 1.8% w/v, and effective collagen I concentration of 0.16% w/v. First, CaCl_2_ (50 mg mL^−1^) was mixed into PBS with concentrations adjusted to the fibrinogen amount (0.06 mg CaCl_2_ ]0.54 mm] per mg fibrinogen). Fibrinogen was then added before tranexamic acid with a concentration of 0.013 mg tranexamic acid per mg fibrinogen. The pH of the blend was adjusted to 8 with 0.7 m NaOH while synthesis was done at room temperature. Then, the cooled collagen solution was gently mixed into the blend and the mixture was cross‐linked with thrombin. The thrombin concentration was adjusted to the fibrinogen fraction to achieve a concentration of 0.185 U thrombin per mg fibrinogen. For a pure fibrin hydrogel, fibrinogen was mixed with PBS, CaCl_2_ (50 mg mL^−1^), and tranexamic acid with final concentrations of 2.5% w/v fibrinogen, 1.04 mg mL^−1^ (9.37 mm) CaCl_2_, and 0.33 mg mL^−1^ tranexamic acid. The pH of the blend was adjusted to 7.4 before cross‐linking with thrombin with a final concentration of 4.63 U mL^−1^ corresponding to the aforementioned concentration of 0.185 U thrombin per mg fibrinogen. The three fibrin‐collagen blends and fibrin hydrogel were synthetized both without and with additional transglutaminase, with a final concentration of 0.32 mg transglutaminase per mg fibrinogen added before tranexamic acid addition. Subsequently, the hydrogel blends were incubated for 3 h prior to experimental analysis. Examples of the mixing protocol for each hydrogel blend can be found in Table S1, Supporting Information, which as well comprised conversion from % to mg mL^−1^ for the hydrogel concentrations. To study the effects of thrombin concentration, blends composed of 1.2% fibrinogen with 0.16% collagen and 1.8% fibrinogen with 0.16% collagen were cross‐linked with 0.06 U thrombin per mg fibrinogen, a third of the aforementioned thrombin concentration. To study the effect of pH during synthesis, a blend comprising 1.2% fibrinogen with 0.16% collagen was synthesized with fibrinogen solution adjusted to pH 7 instead of pH 8 prior to collagen addition. A variant of the blend made of 1.2% fibrinogen with 0.16% collagen was synthetized with additional transglutaminase at the aforementioned concentration, while the blend (pH 8) was cooled to 4 °C for 6 h prior to thrombin cross‐linking (0.185 U thrombin per mg fibrinogen).

### Hydrolytic Stability of Hydrogel Blends: Compaction, Swelling, and Degradation Rate

To study the hydrolytic stability of the hydrogels, four samples per hydrogel blend were prepared with 500 µL each in a 24‐well plate. After cross‐linking, the hydrogel samples were removed and weighed (*m*_0_). The samples were placed in a 12‐well plate and immersed in 1 mL sterile PBS, then incubated. On days 1, 3, 5, 7, 14, 21, and 28 of incubation, the hydrogels were weighed (*m*) and the pH of the PBS was measured while the PBS was changed three times a week. The weight change was compared to the initial weight *m*_0_ and calculated as:

(1) $$\Delta m=\frac{m_{0}-m}{m_{0}}\times 100$$

### Optical Appearance of Hydrogel Blends

To quantify hydrogel opacity and transparency, three samples per hydrogel blend were filled in 96‐well plates with 100 µL each. The absorbance of the gels was measured at 15, 30, 75 min, 6, 18, and 42 h after cross‐linking using a microplate reader (Spectramax M2, Molecular Devices, LLC., San Jose, USA) at 370, 450, 550, and 620 nm. After 75 min, the hydrogel samples were covered with PBS to prevent dehydration, which was aspirated prior to measuring. Data were obtained using the manufacturer's software, Softmax Pro 6.

### Unconfined Compression Tests of Hydrogel Blends

To study the compressive strength of the hydrogel blends, unconfined compression tests were performed. The hydrogels were synthetized in 13‐mL plastic tubes (95 × 16.8 mm, 23160, Sarstedt, Nürnbrecht, Germany) with 12 mL per hydrogel blend. After cross‐linking, sections ≈12 mm in height were cut from the tubes, resulting in five cylindrical hydrogel samples with a diameter (*d*) of 14 mm. The samples were weighed (*m*_0_) and the actual height was measured. Each sample was then tested by compressing with 4 mm min^−1^, a preload force of 0.01 N, and maximum force of 7 N using a universal testing machine (Z2.5, zwickiLine, ZwickRoell GmbH & Co. KG, Ulm, Germany). Data were recorded by the manufacturer's software testXpert II. Samples were weighed again (*m*_1_) after the compression. The weight loss due to compression was calculated as:

(2) $$\Delta m=\frac{m_{0}-m_{1}}{m_{0}}\times 100$$

The stress (*σ*) was calculated by dividing the force (*F*) through the circular cross‐sectional area. The strain was calculated by dividing the height difference of the initial sample height (*H*_0_) and the height *H* through *H*_0_. The Young's moduli for the strain regions of 0–5%, 5–10%, 10–15%, and 15–20% strain were calculated by dividing the stress by the strain.

### Hydrogel Microstructure: Scanning Electron Microscopy

SEM was used to investigate the microstructure of the hydrogel blends. Next to the described fibrin‐collagen blends and pure fibrin gel, pure collagen gel was used for SEM studies as well. Hydrogels were prepared in duplicate and with each 100 µL in 96‐well plates. After cross‐linking and equilibrium swelling overnight, the hydrogels were fixed in 3% v/v glutaraldehyde in 0.1 m Sorensen's phosphate buffer. Samples were dehydrated in ethanol (30%, 50%, 70%, 90%, and 100%) and critical point dried (E‐300 Critical Point Dryer, Polaron Equipment, London, UK). Hydrogels were then sputtered with gold/palladium and imaged using a SEM (ESEM XL 30 FEG, FEI, Philips, Eindhoven, The Netherlands) under a high vacuum at an acceleration voltage of 10 kV.

### Cell Culture

HUVECs were isolated from the endothelium of the veins of human umbilical cords after informed consent approved by the Ethics Committee of the Faculty of Medicine, RWTH Aachen University (EK 424/19). HUVECs were cultivated in T75 cell culture flasks previously coated with 2% w/v gelatin. As a growth medium, endothelial cell basal medium (C‐22111, PromoCell, Heidelberg, Germany) was used and cells were used for experiments between passages two and five.

### HUVEC Proliferation Study, Imaging, and Hydrogel Degradation Due to Cellular Activity

During hydrogel synthesis for the HUVEC proliferation studies, antibiotic‐antimycotic solution (100×) (A5955, Sigma‐Aldrich, Darmstadt, Germany) with a final concentration of 1% v/v was added to the hydrogel blends prior to thrombin cross‐linking. The respective amount was subtracted from the PBS volume. Cell proliferation on hydrogels was investigated over the course of 7 days using the Cell Counting Kit‐8 (CCK‐8 assay, CK04, Dojindo EU, Munich, Germany). Briefly, a water‐soluble tetrazolium salt, WST‐8, was reduced by the cells’ dehydrogenase activities to create a yellow‐colored formazan dye. The amount of dye generated was directly proportional to the number of living cells. The wells of 12‐well plates were loaded with 280 µL of the hydrogel blends. After cross‐linking, HUVECs were trypsinized and counted, and 1 × 10^4^ HUVECs were seeded onto the gels and covered with 686 µL of growth medium. The CCK‐8 assay was performed on days 1, 3, 5, and 7 after seeding. First, old medium was aspirated and the gels were thoroughly washed with PBS to remove any medium residues. The CCK‐8 solution was mixed with HUVEC medium at a ratio of 1:11. Every sample was covered with 370 µL of the CCK‐8 media mixture and the well plates were incubated for 135 min. Next, samples of 100 µL of the mixture were loaded into 96‐well plates, and three blanks with a solution not exposed to cells were also loaded. The optical density of the samples was measured at 450 nm using a microplate reader (see above for specifications). The remaining CCK‐8 media mixture was removed, the gels washed again with PBS, and covered with fresh growth media. In order to calculate the cell multiplication on the respective days, the metabolic activity and number of living cells was normalized to the number of living cells on day 1 (D1) as follows:

(3) $$\mathrm{Fold}\;\mathrm{change}=\frac{\mathrm{OD}-{\mathrm{OD}}_{\mathrm{blank}}}{{\mathrm{OD}}_{D1}-{\mathrm{OD}}_{D1,\mathrm{blank}}}$$

For imaging, the wells of 2‐well slides (µ‐Slide 2 Well Glass Bottom, Ibidi, Gräfeling, Germany) were each loaded with 360 µL of hydrogel blend. After incubation, 1.45 × 10^4^ HUVECs were seeded onto the hydrogels and covered with 1 mL of growth medium to keep the cell seeding density and media volume constant compared to the 12‐well plates. HUVECs were stained using Calcein‐AM (425201, BioLegend, Koblenz, Germany) on days 1, 3, 5, and 7. Briefly, Calcein‐AM was diluted with dimethyl sulfoxide for a stock solution of 1 mm. For a working solution of 1 µm, the stock solution was further diluted with PBS. The staining solution was added to the wells and incubated for 20 min. Dead staining was not performed as preliminary experiments revealed that dead cells would be aspirated during washing and hence could be not be imaged during microscopy. The staining solution was removed, samples were washed with PBS, and images were taken on ten random fields using a fluorescence microscope (Axio Imager.M2m, Carl Zeiss Microscopy Deutschland, Oberkochen, Germany). To assess the normalization of metabolic activity to day 1, ten random images of each hydrogel were analyzed and cells were counted using ImageJ. Experiments were performed with three different donors and three samples per donor. To study the hydrogel degradation by cellular activity, the hydrogels were fixed in 4% formaldehyde (28908, Thermo Fisher Scientific, Waltham, USA) after imaging on the respective days. The hydrogels were then lyophilized (Lyoquest, Telstar, Leeds, UK) and the weight change compared to day 0 was calculated as described in Equation (2).

### Statistical Analysis

Statistical analysis was performed using GraphPad PRISM 9 (GraphPad Software, San Diego, USA). ANOVA tests were performed in order to study significant differences within groups and comparison between groups with Tukey's post‐hoc test. Significance levels of *p* <0.05 (*), *p* <0.01 (**), and *p* <0.001 (***) were defined. Data were presented using box and Tukey whiskers.

## Conflict of Interest

The authors declare no conflict of interest.

## Supporting information

Supporting Information

## Data Availability

The data that support the findings of this study are available from the corresponding author upon reasonable request.
