# Supplementary material for: pH and Thrombin Concentration Are Decisive in Synthesizing Stiff, Stable, and Open‐Porous Fibrin‐Collagen Hydrogel Blends without Chemical Cross‐Linker
Source: Adv Healthc Mater. 2023 Jan 13;12(10):2203302. doi: 10.1002/adhm.202203302 (PMC11468609; doi:10.1002/adhm.202203302)
Supplement: Supplementary file 1 — Supporting Information [file ADHM-12-2203302-s001.pdf]

Supporting Information

pH and thrombin concentration are decisive in synthesizing stiff, stable, and open-porous fibrin-collagen hydrogel blends without chemical crosslinker

*Mattis Wachendörfer, Eva Miriam Buhl, Ghazi Ben Messaoud, Walter Richtering and Horst Fischer**

Table S1: Mixing protocols for 1 ml of the hydrogel blends

**0.6% (6 mg/ml) fibrin 0.16% (1.6 mg/ml) collagen**			
	Substance	Concentration	[ml]
1	PBS		0.33862
2	Fibrinogen	50mg/ml	0.12
3	Transexamic acid	100mg/ml	0.00083
4	CaCl2	50 mg/ml	0.0072
Adjusting pH to 8 using 0.7 M NaOH			
5	Collagen	3.2mg/ml	0.5
6	Thrombin	33.34 U/ml	0.03335

**1.2% (12 mg/ml) fibrin 0.16% (1.6 mg/ml) collagen**			
	Substance	Concentration	[ml]
1	PBS		0.22358
2	Fibrinogen	50mg/ml	0.24
3	Transexamic acid	100mg/ml	0.00163
4	CaCl2	50 mg/ml	0.00144
Adjusting pH to 8 using 0.7 M NaOH			
5	Collagen	3.2mg/ml	0.5
6	Thrombin	66.68 U/ml	0.03335

**1.8% (18 mg/ml) fibrin 0.16% (1.6 mg/ml) collagen**			
	Substance	Concentration	[ml]
1	PBS		0.08262
2	Fibrinogen	50mg/ml	0.36
3	Transexamic acid	100mg/ml	0.00243
4	CaCl2	50 mg/ml	0.0216
Adjusting pH to 8 using 0.7 M NaOH			
5	Collagen	3.2mg/ml	0.5
6	Thrombin	100 U/ml	0.03335

**2.5% (25 mg/ml) fibrin**			
	Substance	Concentration	[ml]
1	PBS		0.34628
2	Fibrinogen	50mg/ml	0.5
3	Transexamic acid	100mg/ml	0.00325
4	CaCl2	50 mg/ml	0.02077
Adjusting pH to 7.4 using 0.7 M NaOH			
5	Thrombin	36 U/ml	0.1297

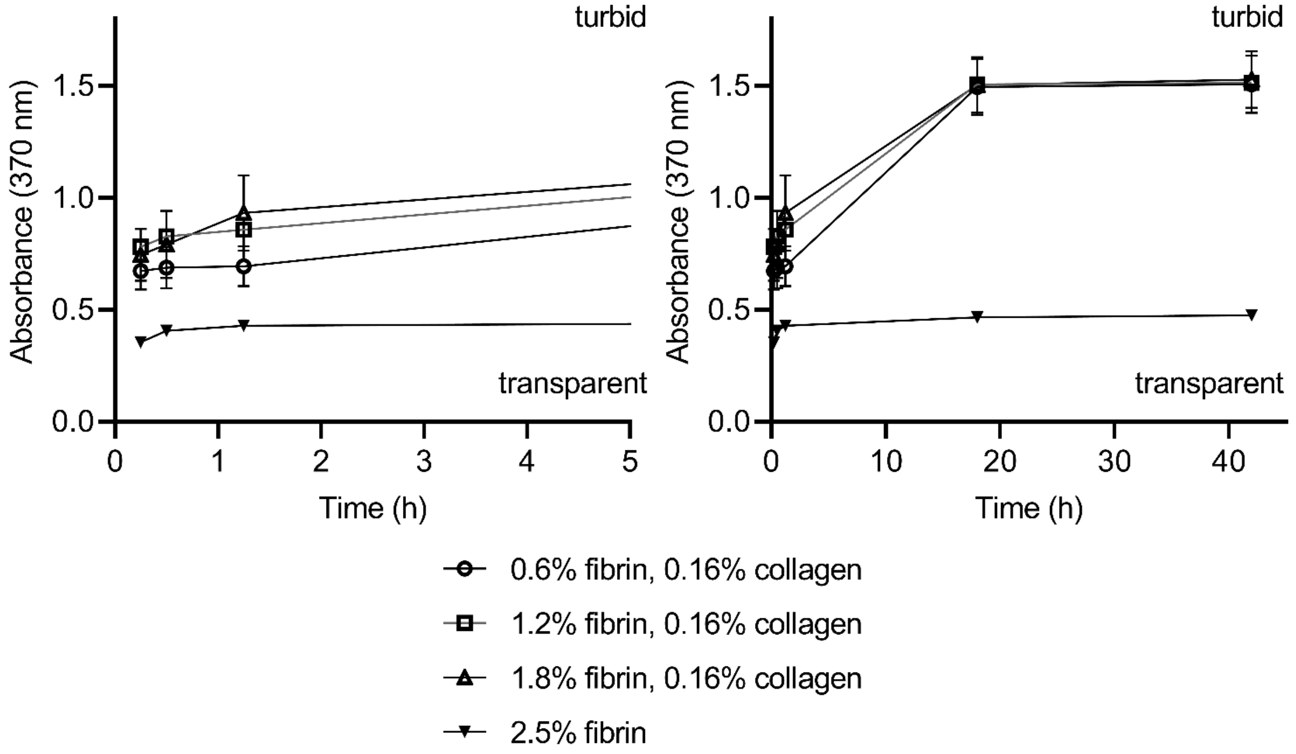

Figure S1. Absorbance at 370 nm of fibrin-collagen blends and fibrin hydrogel. All fibrin-collagen blends and fibrin were synthetized at pH 8 and pH 7.4, respectively, without additional transglutaminase and with a thrombin concentration of 0.185 U thrombin per mg fibrinogen. Measurements of absorbance at 450, 550 or 620 nm revealed identical patterns.

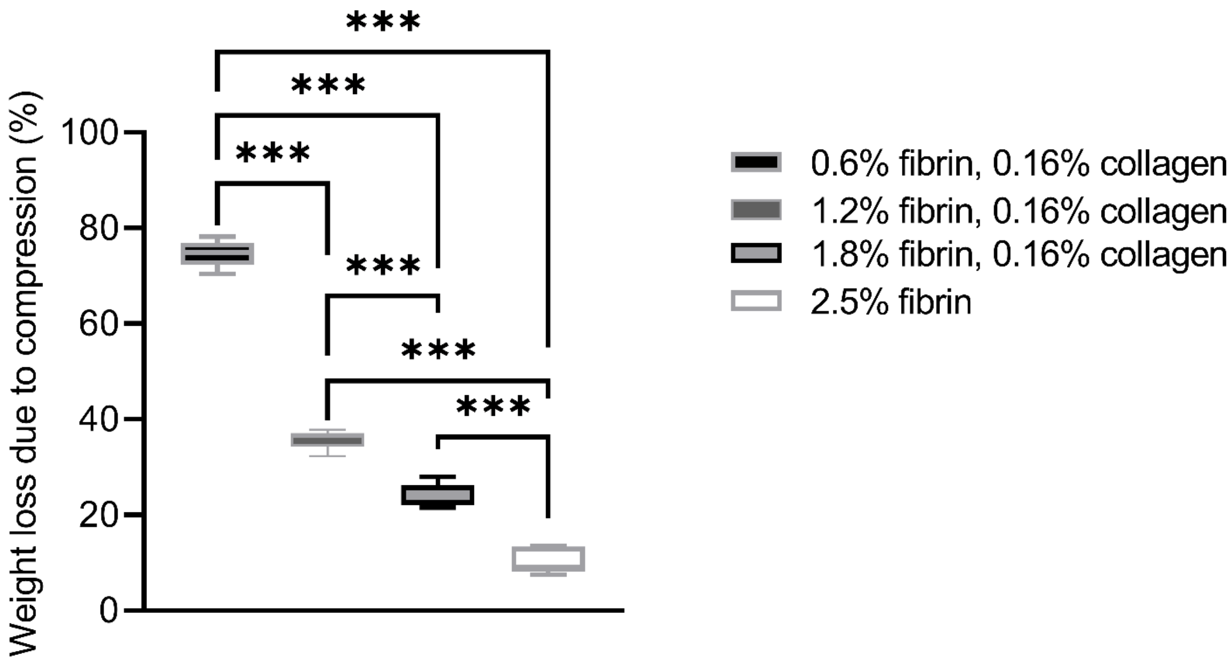

Figure S2. Weight loss due to compression of fibrin-collagen blends. Weight loss significantly decreased with increasing fibrin fraction. All fibrin-collagen blends and fibrin were synthetized at pH 8 and pH 7.4, respectively, without additional transglutaminase and with a thrombin concentration of 0.185 U thrombin per mg fibrinogen.

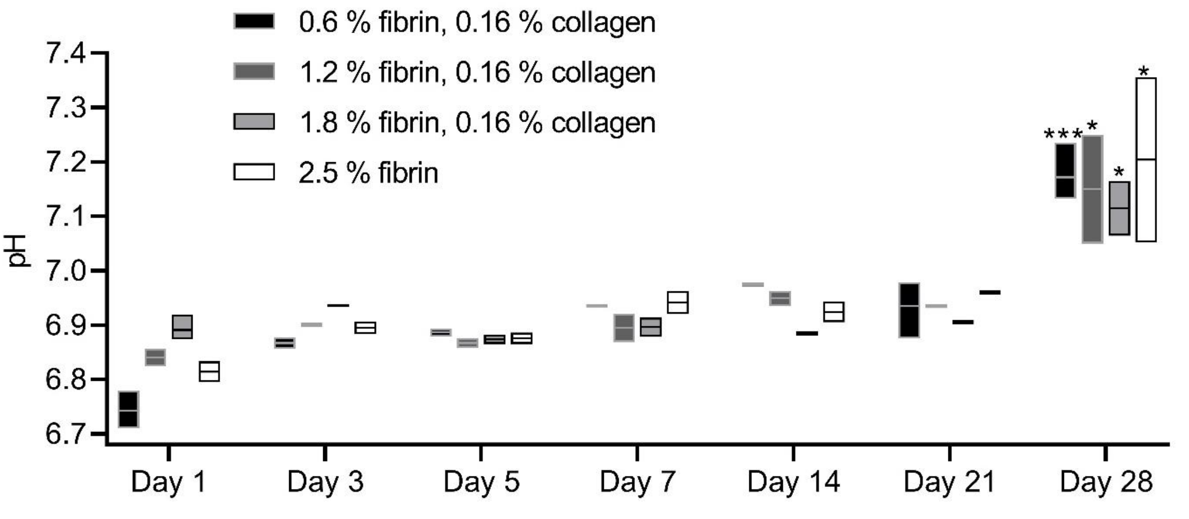

Figure S3. pH value of PBS buffer used for in vitro degradation experiments for fibrin-collagen blends and fibrin. All fibrin-collagen blends and fibrin were synthetized at pH 8 and pH 7.4, respectively, without additional transglutaminase and with a thrombin concentration of 0.185 U thrombin per mg fibrinogen. Significant changes in pH to the previous time point are marked with * or ***. The pH remained constant over the course of 21 days and subsequently increased significantly with advanced degradation from day 21 to day 28.

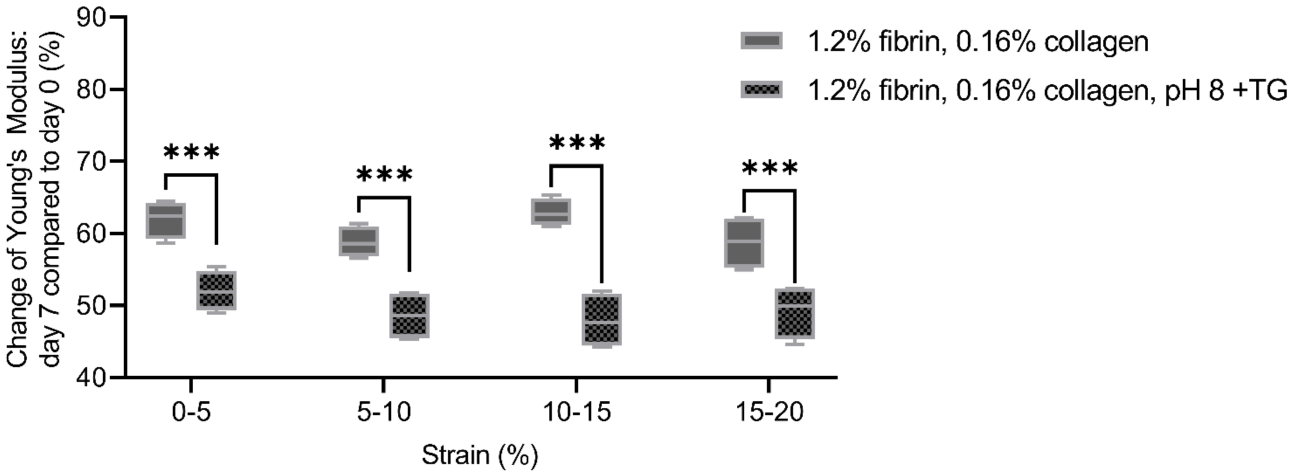

Figure S4. Change of Young's modulus over 7 days of hydrolytic degradation of 1.2% fibrin with 0.16% collagen blends, synthetized at pH 8 without or with transglutaminase (application time 6 h at 4 °C and pH 8 prior to thrombin crosslinking). Blends synthetized with additional transglutaminase but at pH 7 were too soft to be measured after 7 days of incubation. For all blends, a concentration of 0.185 U thrombin per mg fibrinogen was used. When blends were synthetized without transglutaminase, a higher fraction of the initial compressive strength was retained.

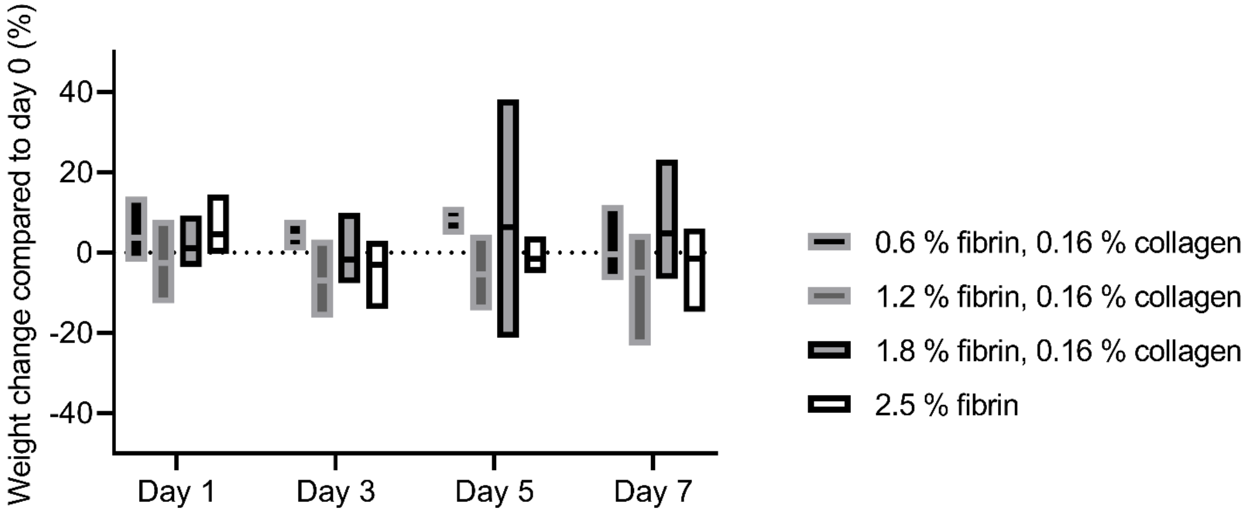

Figure S5. In vitro degradation of HUVEC-laden fibrin-collagen blends and fibrin hydrogel. Following proliferation over 7 days, no significant weight loss of lyophilized blends was found, indicating hydrogel stability during HUVEC proliferation.

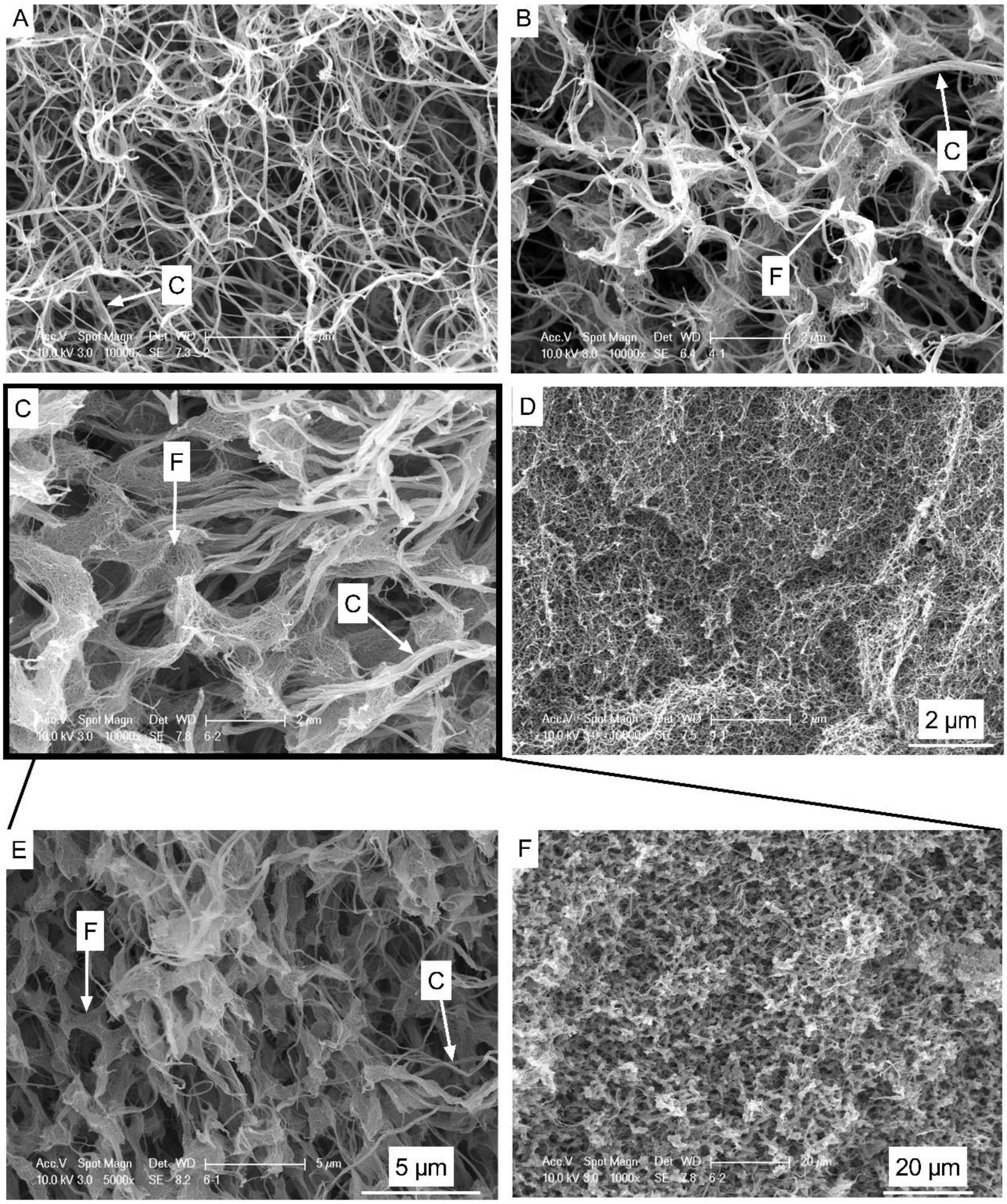

Figure S6. SEM pictures of fibrin-collagen blends and fibrin synthetized with transglutaminase. All fibrin-collagen blends and fibrin were synthetized at pH 8 and pH 7.4, respectively, with additional transglutaminase and with a thrombin concentration of 0.185 U thrombin per mg fibrinogen. A: 0.6% fibrin with 0.16% collagen. B: 1.2% fibrin with 0.16% collagen. C: With 1.8% fibrin with 0.16% collagen blends, depicted at lower magnifications in micrographs E and F. D: Pure 2.5% fibrin gels show a typically dense microstructure. Magnification of A-C identical with D. SEM analysis revealed identical microstructure to blends synthetized without additional transglutaminase depicted in Figure 5, indicating that addition of transglutaminase does not actively crosslink the two components. Fibrin (F) and collagen (C) form an interpenetrating network by molecular entanglement.

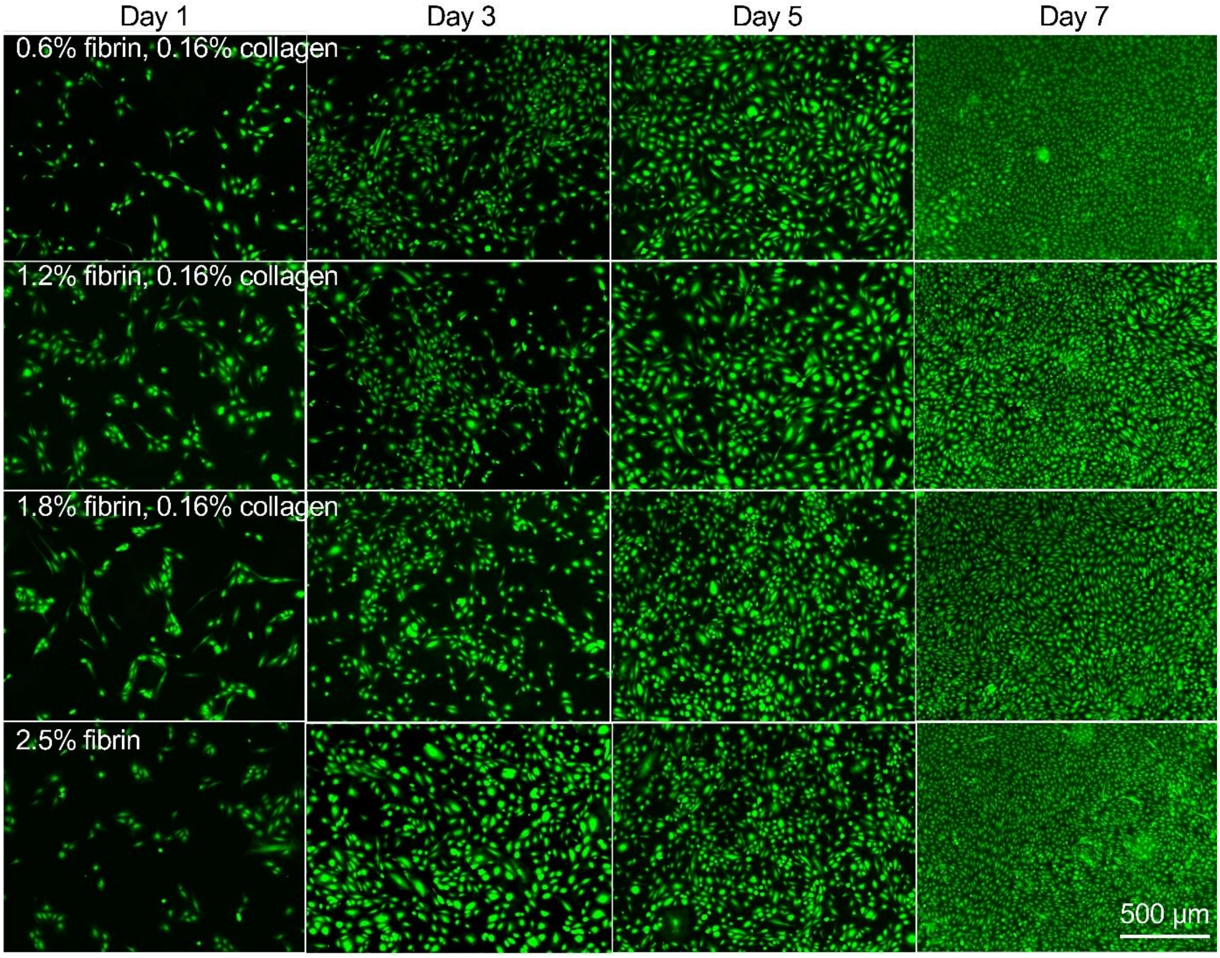

Figure S7. Fluorescence images of Calcein AM stained HUVECs on fibrin-collagen blends and fibrin hydrogel. All fibrin-collagen blends and fibrin were synthetized at pH 8 and pH 7.4, respectively, without additional transglutaminase and with a thrombin concentration of 0.185 U thrombin per mg fibrinogen. After 7 days, a monolayer was formed on all gels. Scale bar identical for every picture.

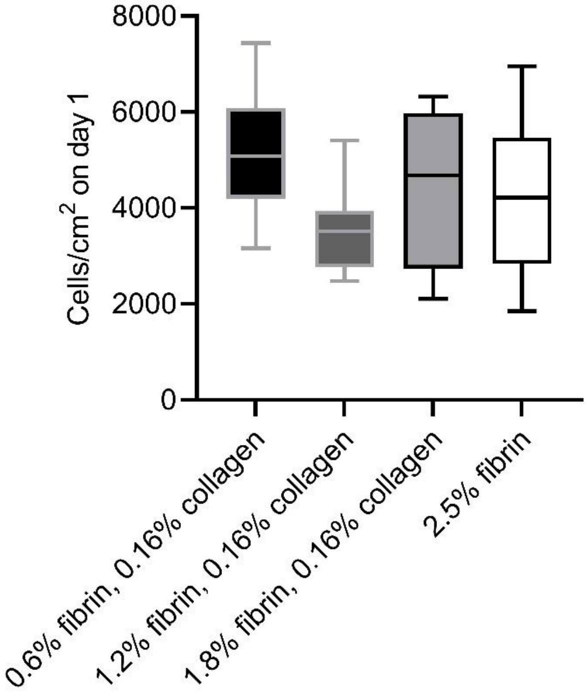

Figure S8. Concentration of HUVECs on fibrin-collagen blends and fibrin hydrogel 1 day after seeding. Cells were counted on fluorescence images (see Figure S6) using ImageJ. No significant differences between the samples were found. Hence, normalization of CCK-8 assay to day 1 (see Figure 6) was performed.

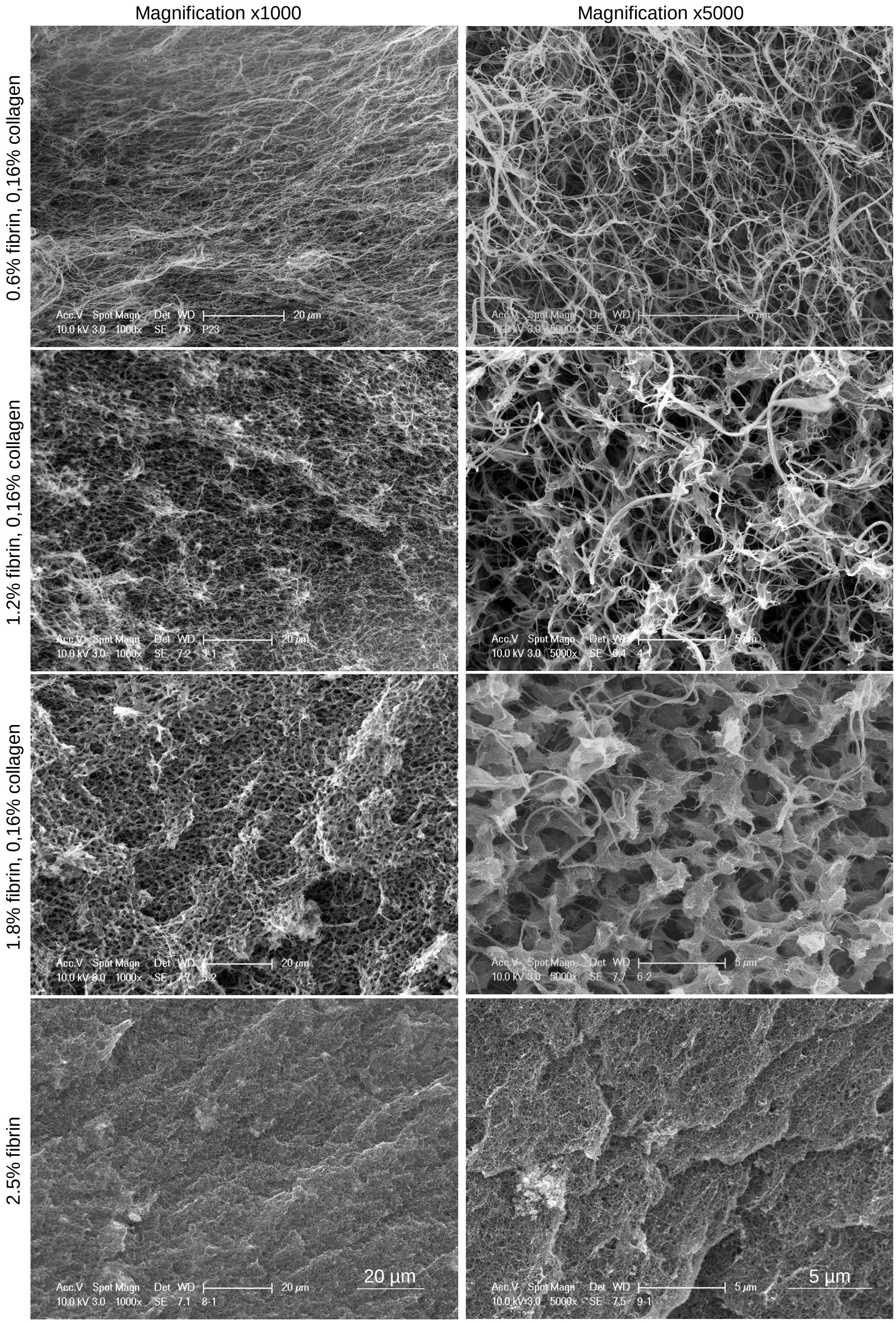

Figure S9. SEM pictures of fibrin-collagen blends and fibrin at x1000 and x5000 magnification. All fibrin-collagen blends and fibrin were synthetized at pH 8 and pH 7.4, respectively, without additional transglutaminase and with a thrombin concentration of 0.185 U thrombin per mg fibrinogen.

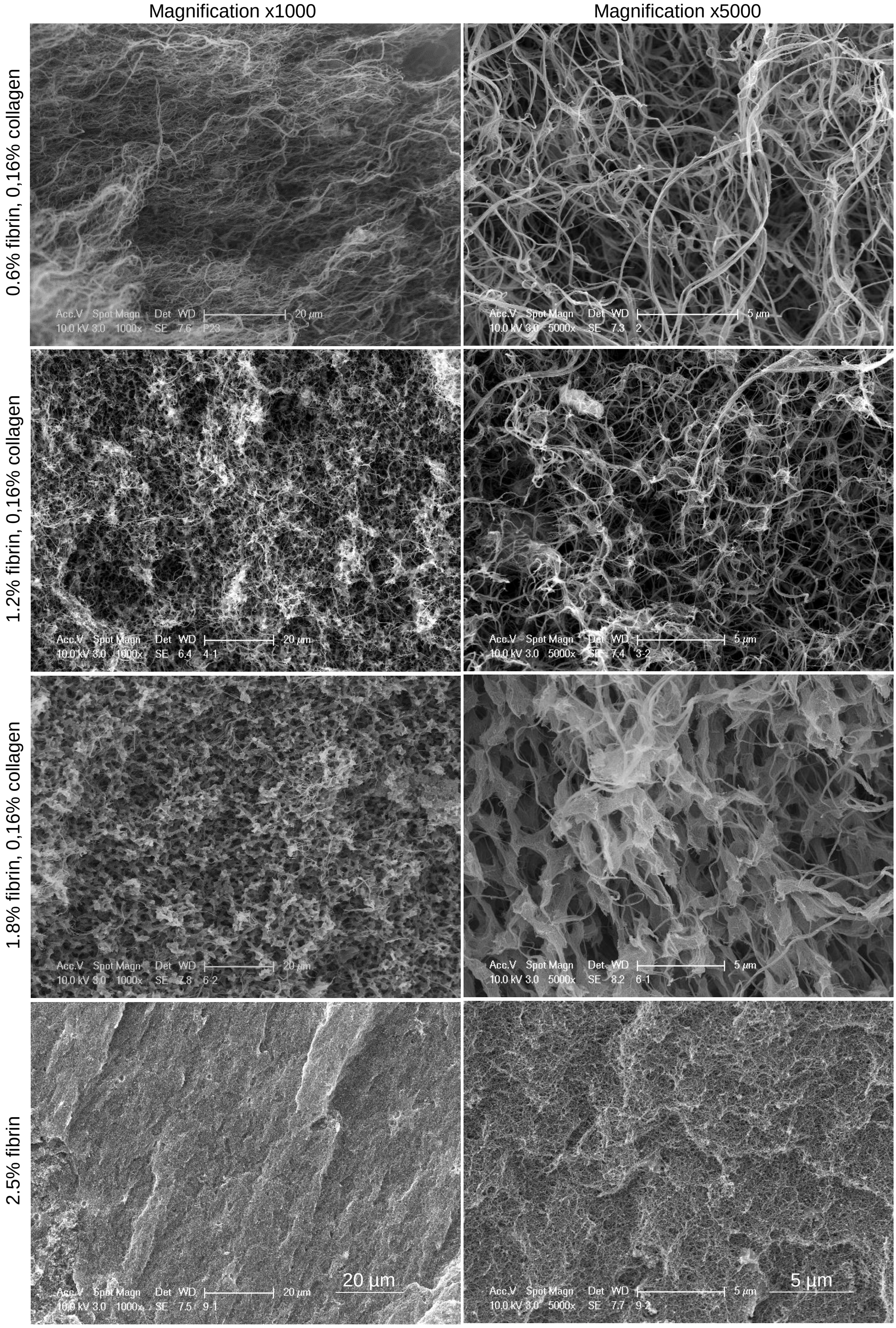

Figure S10: SEM pictures of fibrin-collagen blends and fibrin at x1000 and x5000 magnification. All fibrin-collagen blends and fibrin were synthetized at pH 8 and pH 7.4, respectively, with additional transglutaminase and with a thrombin concentration of 0.185 U thrombin per mg fibrinogen.

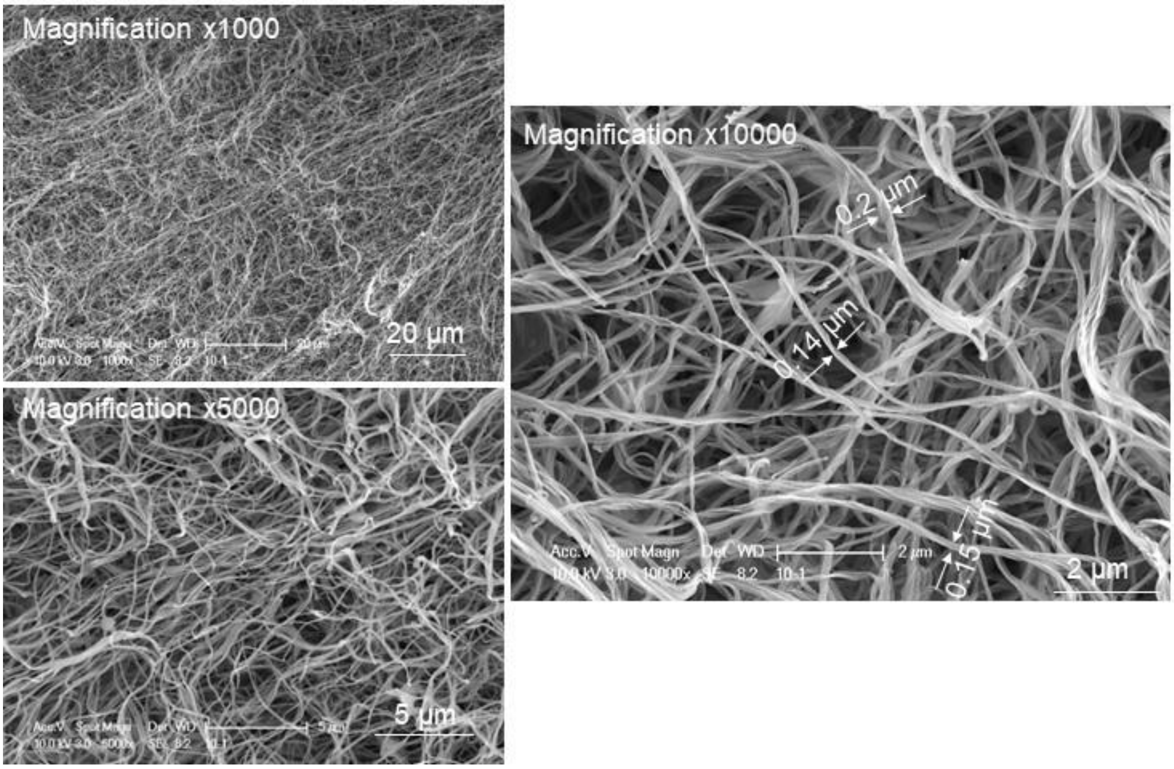

Figure S11: SEM pictures of collagen at x1000, x5000 and x10000 magnification. Collagen showed an arbitrary fiber distribrution with thick fibers, usually intertwined to triple-coiled fiber bundles.
